# Intraventricular Shunt as a Treatment for Entrapped Temporal Horn After Removal of Ventricular Meningioma: A Report of Two Cases

**DOI:** 10.7759/cureus.57561

**Published:** 2024-04-03

**Authors:** Jinhua Xie, Zihuan Zeng, Shousen Wang

**Affiliations:** 1 Department of Neurosurgery, 900th Hospital of the Joint Logistics Support Force, Fuzhou, CHN

**Keywords:** intraventricular shunt, t-connector, hydrocephalus, ventricular trigone tumor, entrapped temporal horn

## Abstract

Entrapped temporal horn (ETH) is a complication following resection of ventricular trigone tumors. It is a special localized hydrocephalus. Obstruction of cerebrospinal fluid outflow following resection of ventricular trigone tumors leads to dilation of the temporal horn and the production of the local space-occupying effect. This article presents two cases of ETH following the resection of ventricular trigone tumors. Our Intraventricular shunt is an effective treatment that uses a T-connector to connect a reservoir with two catheters. We presented temporal-frontal horn shunt and trigone-front horn shunt. A patient presented with ETH shunt dependency. Our intraventricular shunt surgery achieved a good prognosis.

## Introduction

Entrapped temporal horn is a serious complication following resection of ventricular trigone tumors, manifesting as a localized form of hydrocephalus. Obstruction of cerebrospinal fluid from the temporal horn, coupled with continuous cerebrospinal fluid secretion, leads to the dilation of the temporal horn and the production of a local space-occupying effect [1-4]. This can result in related symptoms, such as headache, vomiting, visual impairment, hemiplegia, memory impairment, and decreased consciousness. The main causes include ventricular adhesions, cerebrospinal fluid reflux obstruction due to ventricular wall damage, local inflammation, and postoperative ventricular structural retraction disorder. A challenging aspect is that ETH does not always occur in the acute phase after tumor resection. Current treatment options primarily include external drainage of the temporal horn, ventriculoperitoneal shunt, endoscopic ventriculostomy, and ventricular adhesion lysis [5-6]. This article presents two cases of Intraventricular shunt surgical treatment for ETH after resection of ventricular trigone meningioma. The ETH was connected to different parts of the ventricle through a catheter for the intraventricular shunt, such as the frontal horn and trigone. This method is flexible and has the advantage of less trauma.

## Case presentation

Case 1

**Figure 1:**
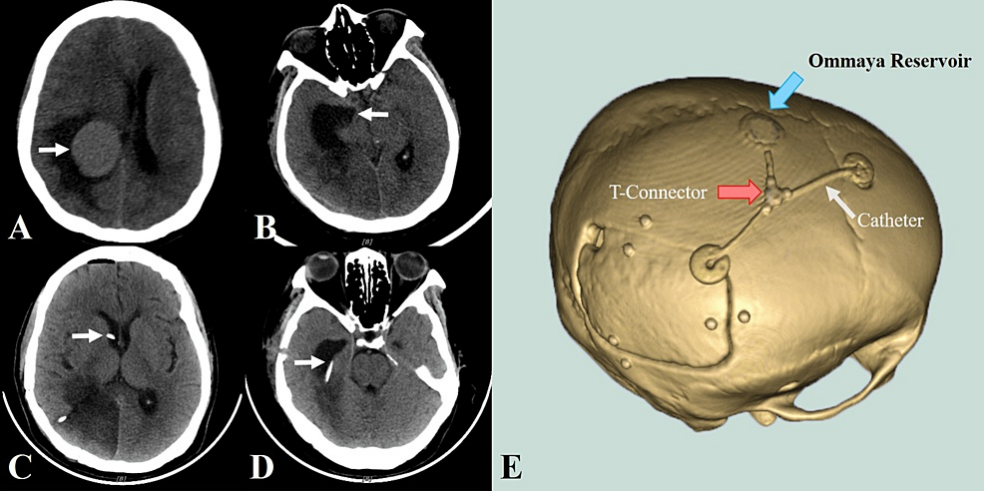
Image of case 1 A: CT scan showed a 4.2 cm × 3.4 cm meningioma in the right trigone with surrounding ventricular edema. B: CT scan demonstrated a temporal horn width of 3.87 cm. C: CT showed the catheter tip on the right frontal horn after the temporal-frontal horn shunt. D: CT showed the catheter tip on the right temporal horn after the temporal-frontal horn shunt. E: 3D reconstruction showed the position of the T-connector, Ommaya reservoir, and extracranial catheter.

**Figure 2:**
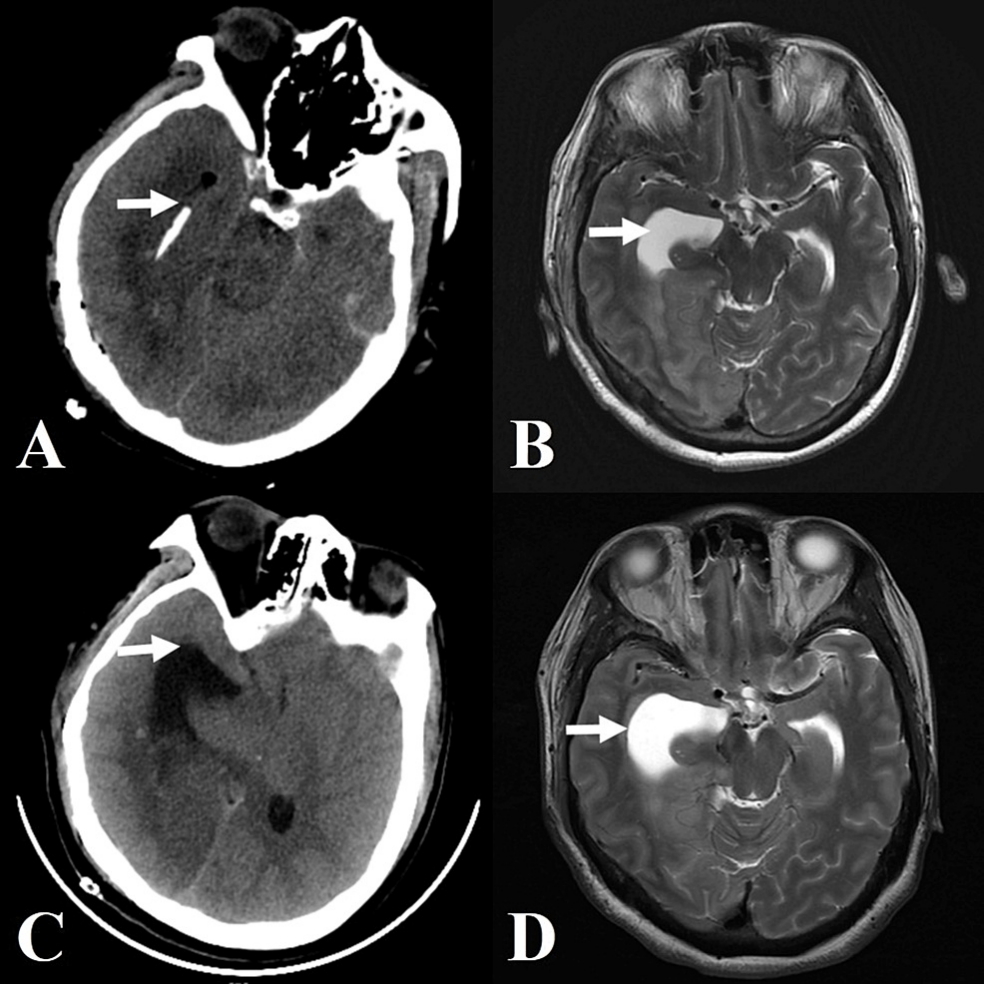
Image of gradual dilation of the temporal horn after surgery in Case 1 A: CT showed temporal horn stenosis on the first day after surgery, along with a narrowed left temporal horn, and the catheter was attached to the ventricle wall. B: MRI showed a right temporal horn width of 3.51 cm on the fourth day after surgery. C: CT scan showed a right temporal horn width of 3.80 cm on the eight day after surgery. D: MRI showed a right temporal horn width of 3.83 cm on the 11th day after surgery.

We present the case of a 47-year-old female who had a sudden headache with disturbance of consciousness for three days, aggravated for five hours. Physical examination upon admission included coma, bilaterally equal-sized pupils, approximately 4 mm in diameter, with delayed direct and indirect pupillary light reflexes. A head CT scan revealed a meningioma next to the right ventricle trigone, measuring 4.2 cm × 3.4 cm. The meningioma secondary cerebral edema in the right cerebral hemisphere, subfalcine hernia, and dilation of the right temporal horn (temporal horn width of 4.31 cm) (Figure 1A-1B). The tumor was resected in blocks, and postoperative pathology confirmed a fibrous meningioma (WHO Grade I). There was no intracranial infection after the operation. A CT scan revealed a 2 ml hematoma in the surgical area on day 1 after surgery. The postoperative image showed gradual dilation of the right temporal horn, with worsening consciousness (Figure 2A-2D). The patient underwent Ommaya reservoir implantation and then underwent ventricular adhesion lysis. However, the patient developed a shunt dependence, manifesting as lethargy and headache upon cessation of drainage. Finally, the patient underwent a right temporal-frontal horn shunt procedure on day 59 of admission. During the surgery, a T-connector (Aesculap-Miethke FV015T) was used to connect the ipsilateral frontal horn, temporal horn, and Ommaya reservoir (Sophysa re-1021) (Figure 1C-1E). Three months after discharge, follow-up imaging revealed a right temporal horn width of 2.71 cm, with the patient remaining neurologically intact, exhibiting good speech and memory function.

Case 2

**Figure 3:**
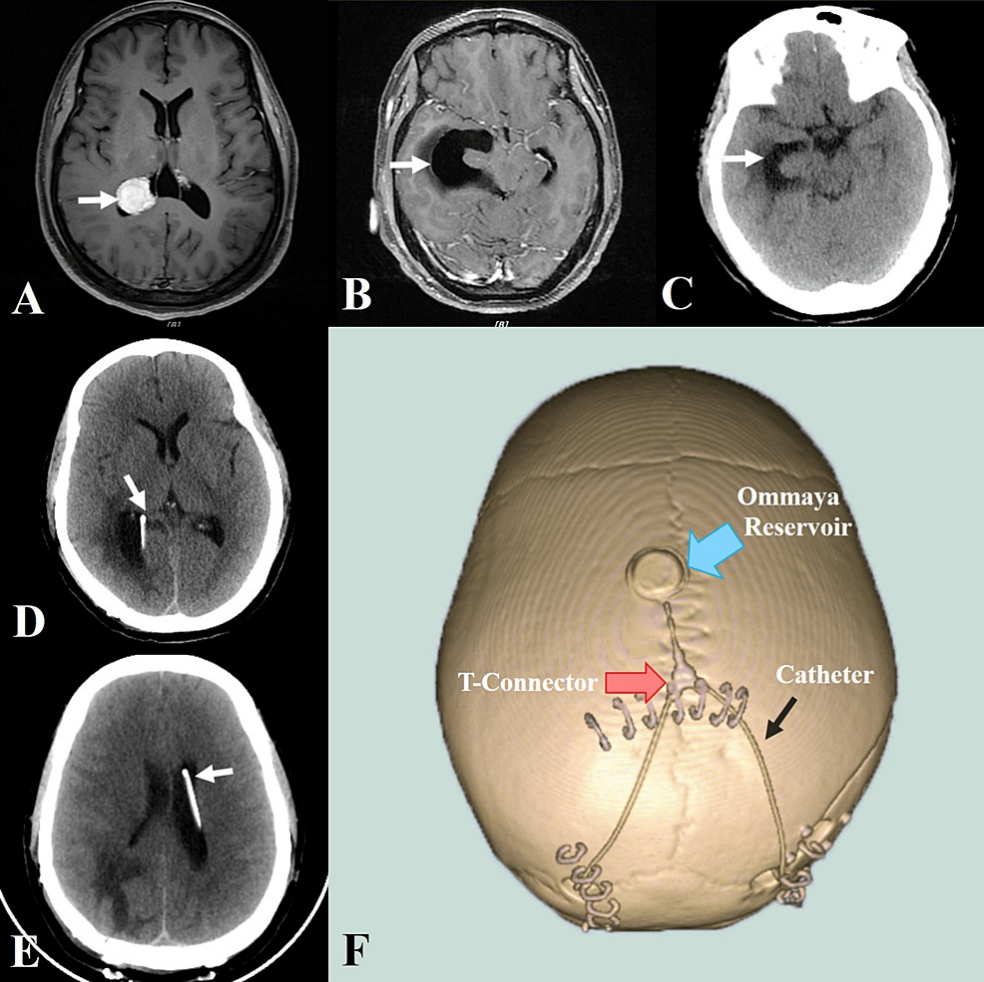
Image of case 2 A: MRI showed a meningioma in the right trigone, measuring 2.1 cm × 2.2 cm. B: MRI demonstrated a right temporal horn width of 3.46 cm on one month after surgery. C: CT showed a right temporal horn width of 2.72 cm on the first day after the intraventricular shunt. D: CT showed the catheter tip on the right trigone. E: CT showed the catheter tip on the left frontal horn. F: 3D reconstruction showed the position of the T-connector, Ommaya reservoir, and extracranial catheter.

We present the other case of a 51-year-old female who had recurrent headaches for four months. Neurological examination upon admission did not reveal any significant abnormalities. MRI demonstrated a meningioma in the right trigone, measuring approximately 2.1 cm × 2.2 cm (Figure 3A). The tumor was resected in blocks, while preserving the ventricular wall intact. Postoperative pathology confirmed a meningothelial meningioma (WHO Grade I). One month after surgery, the patient was readmitted due to headache, dizziness, and quadriparesis. MRI scan revealed significant dilation of the right temporal horn (temporal horn width of 3.46 cm) (Figure 3B). Fifty-five days after the surgery, the patient underwent a bilateral occipital horn centesis for communication between the left frontal horn and the right trigone. A T-connector was used to connect bilateral Ommaya catheters and one reservoir during the surgery (Figure 3D-3F). On the second day after the intraventricular shunt, the CT scan demonstrated a significant shrink in the width of the ETH (Figure 3C), with significant relief of headache and dizziness. The patient was discharged on the 12th postoperative day. Three months after discharge, the patient reported no headache or dizziness. A follow-up brain MRI revealed normalization of the right temporal horn morphology, with no evidence of tumor recurrence.

## Discussion

ETH is a common complication after meningioma surgery in the trigone, which may lead to increased intracranial pressure, cerebral edema, and other related neurological symptoms. To effectively treat this condition, the medical community has explored various surgical approaches, and surgical treatment options are diverse. Currently, there is no unified treatment standard. The intraventricular shunt for the treatment of ETH described in this article adopts an innovative design of connecting two Ommaya catheters and one reservoir with a T-connector. This design allows the cerebrospinal fluid accumulated in the temporal horn to be drained to other appropriate parts of the lateral ventricle, thereby effectively reducing intracranial pressure and relieving cerebral edema. Compared with a traditional ventriculoperitoneal shunt, this method has the advantages of lesser trauma, shorter catheter path, lower operation difficulty, and relatively low cost [7].

In practical applications, we have reported two successful cases of ETH treatment. In one case, the frontal-temporal horn shunt was used to establish a passage between the frontal horn and the temporal horn, allowing cerebrospinal fluid to flow smoothly. In the other case, the occipital horn puncture was performed for the trigone-frontal horn shunt, which achieved decompression by directing cerebrospinal fluid toward the frontal horn. Both intraventricular shunts demonstrated their flexibility and effectiveness.

In Case 1, an Ommaya catheter was placed from the right trigone to the temporal horn during ventricular adhesion lysis. However, due to the narrowness of the right trigone and occipital horn, it was difficult to perform the puncture in this area. Therefore, a frontal horn puncture was performed during the intraventricular shunt, and a T-connector was used to complete the ipsilateral temporal-frontal horn shunt. In Case 2, due to the dilation of the right temporal and occipital horns, which communicated with each other, occipital horn puncture became relatively straightforward. Finally, the prone bilateral occipital horn puncture was performed for the intraventricular shunt. In Case 1, the Ommaya reservoir was vertically implanted into the right temporal horn. Although the surgical difficulty was low, postoperative blockage of the Ommaya reservoir led to re-dilation of the temporal horn and worsening of consciousness, manifesting as shunt dependence. During subsequent ventricular adhesion lysis, the Ommaya reservoir was re-implanted through the trigone, with the catheter placed into the temporal horn, consistent with the modified frontal-temporal horn shunt [8].

After the fourth surgery in Case 1, a frontal horn puncture was performed, and a new catheter was placed. The T-connector connected to the original Ommaya catheter, a new Ommaya catheter, and a new Ommaya reservoir, forming an ipsilateral temporal-frontal horn shunt. The T-connector was placed under the scalp on the right frontal region. This approach not only allowed for the completion of the operation in a single surgical position but also minimized the risk of compressing the tubing while the patient was lying down. In Case 2, a trigone-front horn shunt was performed without a pre-implanted Ommaya reservoir. After adopting a prone position, bilateral occipital horn punctures were performed without taking into account different angles of the ventricle puncture, and this allowed for the completion of the operation in one position.

In Case 1, the meningioma was relatively large, and there was significant edema around the tumor prior to surgery. In addition, there was also dilation of the right temporal horn. Before the emergency surgery, signs of brain herniation and delayed pupillary light reflex were present bilaterally. During the surgery, it was observed that the tumor was partially adherent to the ventricular wall. Combined with the third ventricular adhesion lysis along the surgical tract, it was found that the tension of the brain tissue was high after opening the dura mater. A small amount of hematoma and Surgicel were removed from the surgical area, but no significant adhesion of the ventricular structure was observed. Based on these findings, the possible causes of the ETH in Case 1 may include long-term edema of the ventricular wall and elevated local pressure due to tumor and hydrocephalus, leading to decreased compliance of the ventricle and surrounding brain tissue. In addition, the blockage of cerebrospinal fluid outflow due to hematoma and artificial materials may also be a contributing factor [9-10].

In Case 2, there was no significant shift in the surrounding ventricular tissue before and after surgery. On the first day after meningioma surgery, a CT scan revealed the presence of a small hematoma in the surgical area. Two weeks later, a brain MRI still showed a signal indicating the presence of hematoma, which might be due to a combination of hematoma and Surgicel. Initially, this mixture partially blocked the cerebrospinal fluid outflow from the temporal horn, ultimately resulting in a complete blockage of the trigone and ETH.

## Conclusions

The surgical treatment strategy for ETH requires personalized selection based on the specific situation of each patient. By flexibly applying different surgical techniques and making innovative use of T-connectors, we can more effectively treat ETH. By considering the specific characteristics of each patient's condition and utilizing appropriate surgical techniques, we can effectively manage ETH and improve patient outcomes.
